# Toxigenic Potential of *Aspergillus* Species Occurring on Maize Kernels from Two Agro-Ecological Zones in Kenya

**DOI:** 10.3390/toxins4110991

**Published:** 2012-10-25

**Authors:** Sheila Okoth, Beatrice Nyongesa, Vincent Ayugi, Erastus Kang’ethe, Hannu Korhonen, Vesa Joutsjoki

**Affiliations:** 1 School of Biological Sciences, University of Nairobi P. O. Box 30197–00100 Nairobi, Kenya; Email: bmutele@yahoo.com (B.N.); vayugi811@gmail.com (V.A.); 2 Department of Public Health Pharmacology and Toxicology, University of Nairobi 30197-00100 Nairobi, Kenya; Email: mburiajudith@gmail.com; 3 Biotechnology and Food Research, MTT Agrifood Research, 31600 Jokioinen, Finland; Email: hannu.j.korhonen@mtt.fi (H.K.); vesa.joutsjoki@mtt.fi (V.J.)

**Keywords:** *Aspergillus*, section *Flavi*, aflatoxins, maize, Kenya

## Abstract

Two agro-ecological zones in Kenya were selected to compare the distribution in maize of *Aspergillus *spp. and their toxigenicity. These were Nandi County, which is the main maize growing region in the country but where no human aflatoxicoses have been reported, and Makueni County where most of the aflatoxicosis cases have occurred. Two hundred and fifty-five households were sampled in Nandi and 258 in Makueni, and *Aspergillus* was isolated from maize. *Aspergillus flavus* and *A. parasiticus* isolates were tested for the presence of *aflD *and *aflQ *genes. Positive strains were induced to produce aflatoxins on yeast extract sucrose and quantified using liquid chromatography-tandem mass spectrometry (LCMSMS). *Aspergillus flavus* was the most common contaminant, and the incidence of occurrence in Nandi and Makueni was not significantly different (82.33% and 73.26%, respectively). Toxigenic strains were more prevalent than non-toxigenic strains. All the toxigenic strains from Makueni were of the *S*-type while those from Nandi belonged to the L-type. Quantitative differences in aflatoxin production *in vitro* between isolates and between strains were detected with S strains producing relatively larger amounts of total aflatoxins, B toxins and lower values for G toxins. This was in accord with the frequent aflatoxicosis outbreaks in Makueni. However some L strains produced considerable amounts of B toxins. Given the widespread distribution of toxigenic strains in both regions, the risk of aflatoxin poisoning is high when favorable conditions for toxin production occur.

## 1. Introduction

Maize is a staple food crop in Kenya for both urban and rural areas with an estimated 1.6 million hectares under cultivation. It is grown by both small- and large-scale farmers. Small-scale farmers contribute 75% of the total maize produced in the country. The quantity of maize consumed in Kenya per person per year is approximately 98 kg translating to about 30–34 million 90 kg bags per year [1], and in some families maize may be consumed twice daily. Each family in Kenya has a garden if not a farm where they grow maize that is mostly for their own consumption and sometimes for sale signifying the importance of this crop in the country. Several cases of aflatoxicosis have been reported in Kenya yearly since 1981 following consumption of maize contaminated with *Aspergillus flavus* and aflatoxins. In 1981 the outbreak was as a result of drought followed by heavy rains during harvest of home grown maize [2]. The worst outbreak was in 2004 where 317 cases and 125 deaths were reported [3], and ever since cases have been reported yearly. In 2010 Kenya had about 2.3 million bags (estimated at $69 million) of maize contaminated with aflatoxins making it unfit for both human and livestock consumption and also for trade, which was a loss to the small-scale farmers who depend on the crop for food and income [4].

*Aspergillus flavus *and *A. parasiticus *are ubiquitous and cosmopolitan fungi producing aflatoxins on a wide variety of substrates such as maize, peanut and cotton. *Aspergillus flavus* is a very important toxigenic fungus that produces aflatoxins that are toxic and of great concern because of their health effects on humans and animals [5]. *Aspergillus *can attack crops at different times, in the field, during harvest, transport and storage. High moisture and temperatures are favorable for the growth of this fungus and toxin production. 

In this study two agro-ecological zones were selected to compare the distribution in maize of *Aspergillus *spp. and their toxigenicity. The Nandi district in the Rift Valley Province was chosen as it is the main maize growing zone in the country, but no aflatoxicosis has been reported in this region. The other zone is the Makueni district in the Eastern Province where most aflatoxicosis cases have been reported. 

## 2. Materials and Methods

### 2.1. Description of Study Sites

Nandi County lies in the Rift Valley Province at elevations between 900–1800 m with an annual rainfall of between 950 and 1500 mm, a mean temperature of 20 °C and one maize planting season from March to April. Makueni County, on the other hand, is in a semi-arid zone in the Eastern Province at an elevation between 800–1700 m with an annual rainfall of between 300 and 600 mm and a mean temperature of 24 °C. Makueni County has two maize planting seasons, from March to May and from October to December. Across the regions from Nandi to Makueni, mean temperatures increase as rainfall decreases [6].

Three sublocations were selected for sampling from each of the two Counties. This study is part of a larger project whose aim is to survey aflatoxin exposure in the maize value chain. The selection of sublocations for the study in this project was based on rearing of dairy cattle and maize cultivation. Kilibwoni, Kaptumo and Laboret were selected in Nandi County while Ukia, Nguumo and Wote locations were selected in Makueni County. Within the sublocations, households that qualified for sampling were those that had children below five years of age. Such households were listed, and random samplings were carried out to select the required sample size, which was calculated using the formula described by Cochrane [7]. From August to December 2010, 255 households were sampled in Nandi County and 258 households in Makueni County.

### 2.2. Sample Collection

Five hundred grams of shelled maize kernels were collected randomly in sterile paper bags from the household storage facilities within the sublocations, sealed and stored at 4 °C. Maize stored in sacks was sampled from different parts using a closed spear driven through the top and sides of each sack to obtain a total of 500 g of incremental samples. In stores where there were less than 10 sacks, all were sampled, while for those storing over ten to one hundred, ten sacks were randomly sampled [8].

### 2.3. Isolation and Identification of Moulds

Five kernels were surface sterilized for 1 min in 2.5% NaOCl, washed in three changes of sterile distilled water and plated on ¼ strength Potato Dextrose Agar (PDA) prepared from Potato Dextrose Broth (Difco) amended with 2 mL/L lactic acid to suppress bacterial contamination. Six replicates from each household were plated. Plated kernels were incubated at 31 °C for three days. Fungal growth colonies on maize kernels were visualized using stereo-binocular microscope (Magnus M24), counted and identified to genus level according to the following authorities: *Fusarium* spp. according to Nelson *et al.* [9]; *Aspergillus* spp., *Penicillium* spp., and other fungi according to Pitt and Hocking [10]. Representative isolates of fungal species that could not be identified directly were transferred onto PDA and those suspected to be *Fusarium* were also grown on Spezieller Nahrstoffarmer agar (SNA) and carnation leaf agar medium to confirm the genus using the description by Leslie and Summerell [11]. Only *Aspergillus *species were single spored and transferred onto PDA medium to study macro and micro morphological characteristics for identification using the taxonomic keys of Klich [12]. These were then transferred onto *Aspergillus flavus parasiticus* agar (AFPA) as described by Pitt *et al.* [13], and incubated in the dark at 28 °C for 42 to 72 hrs to confirm group identification by colony reverse color. The isolates were also grown on 5/2 agar medium (5% V-8 juice and 2% agar, pH 5.2) at 30 °C for 7–10 days to induce sclerotia formation for strain classification [14]. 

### 2.4. Molecular Characterization

#### 2.4.1. Detection of Aflatoxin Genes *aflD (=nor1)* and *aflQ (=ordA = ord-1)*

The conventional naming system of the aflatoxin genes outlined by Yu *et al.* [15] is used in this report. All *Aspergillus flavus* isolates (78 from Nandi; 87 from Makueni) and *A. parasiticus* isolates (22 from Nandi; 7 from Makueni) were tested for presence of *aflD*, and *aflQ *genes. The *aflD* gene encodes an enzyme that catalyzes the conversion of the first stable aflatoxin biosynthesis intermediate, norsolorinic acid to averantin in both *A. flavus* [16] and *A. parasiticus* [17] while the *aflQ* gene is involved in the conversion of *O*-methylsterigmatocystin (omst) to aflatoxin B_1_ (AFB1) and aflatoxin G_1_ (AFG1) and dihydro-*O*-methylsterigmatocystin (dmdhst) to aflatoxin B_2_ (AFB2) and aflatoxin G_2_ (AFG2) in *A. parasiticus* [18] and in *A. flavus* [15] 

#### 2.4.2. DNA Extraction

A spatula full of spores was transferred from a seven-day old culture into a 2 mL eppendorf tube containing 2 mm diameter glass beads, then 650 µL of lysis CTAB buffer was added and the mixture was ground in a tissue miller at a frequency of 30/sec for 3 minutes after which the samples were incubated at 65 °C for one hour. Proteins were precipitated by adding 600 µL phenol inverted gently and centrifuged at 14000 rpm for 20 minutes. The top aqueous layer was pipetted to a new tube, 600 µL phenol:chloroform (25:24 v/v) was added, inverted gently and centrifuged at 14000 rpm for 20 minutes. This procedure was repeated twice with 600 µL of chloroform. Clean supernatant was transferred to a new 1.5 mL eppendorf tube to which 60 µL 3M sodium acetate (pH8) and 800 µL isopropanol were added, inverted gently and incubated overnight at 4 °C for precipitation. The samples were centrifuged at 14000 rpm for 10 minutes at 4 °C, the supernatant discarded, after which the DNA pellet was washed twice with 1 mL 70% ethanol and centrifuged again at 14000 rpm for 5 minutes at 4 °C. The supernatant was discarded and the DNA pellet dried in an oven with the eppendorf tube lid open at 55 °C for 30 minutes. The pellet was re-suspended in 80 µL low salt TE buffer, 5 µL RNase (1mg/mL) was added to remove any RNA contamination and, depending on the yield after running 1 µL on 1% agarose gel, stored at −20 °C.

#### 2.4.3. PCR Amplification

Polymerase chain reaction (PCR) amplifications were performed on 25 µL of a reaction mixture containing MgCl_2_-free reaction buffer, 50 mM MgCl_2_, 10 mM dNTP mix, 10 µM of each primer, 5 U/µL Taq DNA polymerase and 5 ng/µL of template DNA. PCR was carried out as follows: (1) one step at 94 °C for 3 minutes; (2) 30 cycles of the following three steps: 1 minute at 94 °C, 1 minute at 57 °C, 1 minute at 72 °C and (3) one final 10-minute step at 72 °C.

The alfD gene was tested using the primers Nor1-F (5′-ACC GCT ACG CCG GCA CTC TCG GCA C-3′) and Nor1-R (5′-GTT GGC CGC CAG CTT CGA CAC TCC G -3′) developed by Rodrigues, *et al.*, [19]. The *aflQ *gene was tested using the primers Ord1-gF (5′-TTA AGG CAG CGG AAT ACA AG-3′) and Ord1-gR (5′-GAC GCC CAA AGC CGA ACA CAA A-3′) [20]. 

### 2.5. Aflatoxigenicity of the Isolates

#### 2.5.1. Fluorescence on Coconut Agar Medium (CAM)

Isolates that were positive for either *aflD* or *aflQ* or both, were inoculated on coconut agar medium (CAM) to detect aflatoxin production [21]. Four negative isolates from each region were also inoculated on CAM to act as controls. The plates were incubated at 30 °C for 7 days then examined under UV light (365 nm) after 3, 5 and 7 days.

#### 2.5.2. Aflatoxin Analysis

Sixty-five percent of *A. flavus* isolates that were positive for either *aflD* or *aflQ* or both and fluoresced on CAM were randomly selected and grown on aflatoxin-inducing Yeast Extract Sucrose (YES) Agar [19] to test their aflatoxin production profile. All the positive *A. parasiticus* isolates were also included. The isolates were inoculated on 9 cm diameter Petri plates and incubated at 27 °C for 7 days in the dark. Aflatoxins were extracted using the methods described by Vega [22] and Smedsgaard [23] with some modifications. Using a 9 mm diameter sterile cork borer, 9 plugs were harvested uniformly from the plates into 50 mL propylene tubes. Three plugs were randomly picked and added to a 5 mL amber screw-cap vial of known weight and weighed again. The plugs were extracted in 2 mL methanol:formic acid (25:1) for 1 h at 25 °C ultrasonically. Using a sterile syringe, the extract was drawn from the vials and filtered through 0.2 µm nylon membrane filter discs into a clean autosampler vial (11.6 mm OD × 32 mm height) and analyzed by liquid chromatography-tandem mass spectrometry (LCMSMS) (Xevo^TM^ Waters). Aflatoxin standards were supplied by Sigma (South Africa Part number 46300-U). A mix of aflatoxins contained 1000 µg/kg of AFG1 and AFB1 and 3000 µg/kg of AFB2 and AFG2. This mixture was then diluted 10×, 100× and 1000× to create four calibration standards (including the undiluted mix) (1 µg/kg, 10 µg/kg, 100 µg/kg, 1000 µg/kg). These four standards were injected separately onto the LCMSMS, and a calibration curve was created for each of the four aflatoxins (peak area *vs.* concentration). The injection volume was 10 µL for the aflatoxin standard mix and 5 µL for all the other standards and the samples. The sample concentrations were determined from the area of each of the aflatoxins using the calibration curve of each aflatoxin. The aflatoxin concentration in the Petri dishes was obtained using the equation below:

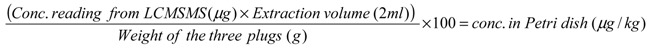



### 2.6. Statistical Analysis

Statistical analysis was done using the program R [24]. Qualitative and quantitative binary Chi square tests for equality of proportions (Pearson's Chi-squared test with Yates' continuity correction) were used to compare frequencies of occurrence of isolates. 

## 3. Results

### 3.1. Fungal Profile of Maize Kernels from Nandi and Makueni Districts

The internal mycoflora associated with maize kernels after surface sterilization is shown in Table 1. Fungi grew from all Makueni samples, but only from 11% of the Nandi samples. None of samples from the Laboret location grew any fungus up until the 10th day of incubation. Nandi recorded a significantly higher (*χ*^2^_4,0.05_ =16.15, *p* = 0.0028) incidence of fungal growth from the kernels compared to Makueni, Table 1. *Aspergillus* was the most common fungus isolated from maize in both regions with over 60% incidence in all locations followed by *Fusarium* species and *Penicillium*. Other genera were *Drechslera*, *Curvularia*, *Rhizopus* and *Alternaria*. Variations in contamination were also observed within the regions with Kaptumo in Nandi and Ukia in Makueni having the highest fungal incidence (*χ*^2^_4,0.05 _= 65.4; *p* < 0.0001). Within the locations, *Aspergillus* was still the predominant genus and the incidence of occurrence of all the other genera varied with locations. In Nandi, *Aspergillus* was predominant in Kaptumo while *Fusarium* and *Penicillium* was more common in maize from Kilibwoni. In Makueni, *Aspergillus* was predominant in samples from Nguumo while *Fusarium* and *Penicillium* were frequently isolated from Ukia samples.

**Table 1:** Frequency of isolation of fungi from maize kernels in locations in two regions in Kenya.

Region	Fungal genera					
	*Aspergillus* sp.	*Fusarium* sp.	*Penicillium* sp.	*Trichoderma* sp.	Others	Total
**Makueni**	189 (62)	71 (23)	25 (8)	10 (3)	12 (4)	307(100)
Nguumo	70 (73)	16 (17)	4 (4)	1 (1)	5 (5)	96 (100)
Ukia	82 (52)	48 (31)	17 (11)	7 (4)	3 (2)	157(100)
Wote	37 (69)	7 (13)	4 (7)	2 (4)	4 (7)	54 (100)
**Nandi**	203 (67)	50 (16)	18 (6)	6 (2)	29 (9)	306 (100)
Kaptumo	94 (73)	16 (13)	6 (5)	0 (0)	12 (9)	128 (100)
Kilibwoni	58 (54)	27 (25)	9 (8)	6 (6)	7 (7)	107 (100)
Laboret	51 (72)	7 (10)	3 (4)	0 (0)	10(14)	71 (100)
**Total (Nandi + Makueni)**	392 (64)	121 (20)	43 (7)	16 (3)	41 (6)	613 (100)

(1). The values are frequencies while those in brackets are row percentages. (2). The frequencies of isolating different genera varied significantly with regions (Chi sq = 16.15, df = 4, *p* = 0.0028). (3). The frequencies of isolating different genera varied significantly with location (Chi sq = 65.4, df = 20, *p* < 0.0001).

### 3.2. Population Composition of the Genus *Aspergillus*

*Aspergillus* was present in 80% (*n* = 203) and 73% (n = 189) of the samples from Nandi and Makueni, respectively. This variation was not significant (χ^2^_0.05(1)_ = 1.885; *p*-value = 0.1698). However, there was evidence of variation in *Aspergillus* presence in maize among the locations (χ^2^_0.05(5)_ = 50.5662; *p*-value = 1.061e-09).

**Figure 1:**
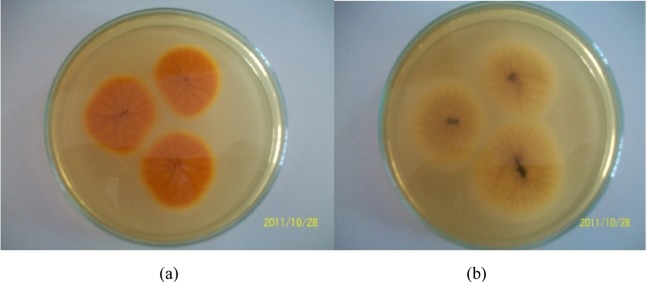
*Aspergillus *isolates grown on *Aspergillus flavus parasiticus* agar (AFPA) medium to identify members belonging to Section *Flavi*; (a) Positive isolate (b) Negative isolate.

Members of Section *Flavi*, identified with a bright orange reverse color on the AFPA medium (Figure 1a), were the most dominant species occurring in maize constituting 60% of the total isolates. This was followed by Section *Nigri* (27%) Section *Fumigati* (9.7%), Section *Circumdati* (3%) and Section *Clavati* (0.3%), Negative isolates had either cream or black reverse colors as shown in Figure 1b. 

Within the locations the highest incidence of Section *Flavi* was recorded in Nandi and the lowest in Makueni (Table 2). Three species, *A. tamarii*, *A. flavus *and *A. parasiticus* were all recovered from maize kernels. *Aspergillus flavus* was the most prevalent in both Nandi and Makueni (58.3%; *n* = 78; 82.8%; *n* = 87, respectively), followed by *A. tamarii *(25.8%; *n* = 33; 10.8%; *n* = 10) and *A. parasiticus* (15.9%; *n* = 21; 6.6%; *n* = 6). All the three species frequently occurred singly. Co-occurrence was observed between *A. tamarii* and *A. flavus* in eight households and *A. flavus* and *A. parasiticus *in three households. *A flavus* was predominant in Makueni maize. *Aspergillus parasiticus* and *A. tamarii* were frequently isolated from Nandi than Makueni.

Morphological characteristics of *A. flavus* and *A. parasiticus* on AFPA and V8-juice was used to differentiate the two species. *Aspergillus flavus* had yellow green colonies, was predominantly biseriate or biseriate and uniseriate with smooth to finely rough conidia; while *A. parasiticus* had dark green colonies with a diameter of between 24–36 mm, was predominantly uniseriate or uniseriate with 20% biseriate and had rough conidia. *Aspergillus tamarii* had dark brown colonies with a diameter of 2–10 mm, was uniseriate with spiky globose conidia. The reverse of *A. tamarii* was bright orange in early stages and turned dark brown after four days. 

All the isolates were grown on V8-juice agar for sclerotia formation. Sclerotia formed on the surface of the agar after 3–7 days of inoculation. Sclerotia formation was more frequent from Makueni *A. flavus* isolates (71%; *n* = 61) compared to Nandi (37%; *n* = 29). Further, Makueni isolates were all of the S-type producing sclerotia with a diameter range of 200–320 µm. Nandi isolates on the other hand were all L-type producing sclerotia of a diameter range of 1000–3100 µm. Sclerotia formed by *A. parasiticus* isolates from Nandi (935–1590 µm) were larger than those of Makueni isolates (240–280 µm). *Aspergillus tamarrii* did not form sclerotia on V8-juice agar (Table 2). 

**Table 2:** Distribution and characteristics of *Aspergillus* Section *Flavi* in two agro-ecological zones, Kenya.

	Location	Number of isolates	*A. flavus* (%)	Strain	*A. parasiticus* (%)	*A. tamarii* (%)
Nandi	Kilibwoni	33	64	L	21	15
	Kaptumo	65	55	L	9	36
	Laboret	34	62	L	26	12
	No. producing sclerotia (%)		37 (n = 29)		19 (n = 4)	0
Makueni	Nguumo	41	95	S	5	0
	Ukia	42	67	S	7	26
	Wote	20	94	S	6	0
	No. producing sclerotia (%)		71(n = 61)		67 (n = 4)	0

**Figure 2:**
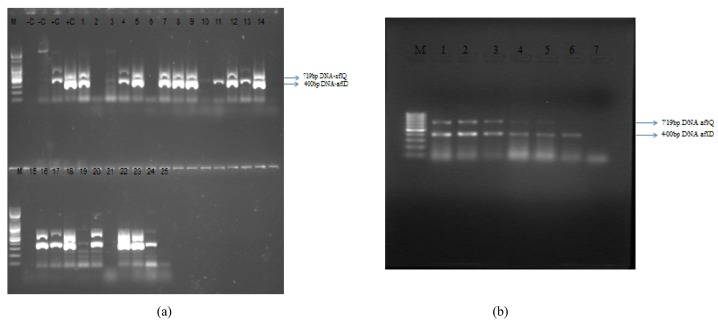
Agarose gel electrophoretic pattern of polymerase chain reaction (PCR) products expressing aflD and aflQ genes. (a) *Aspergillus flavus* isolates from Makueni county : M—molecular weight 100 bp ladder (Promega); -C—Negative control 3VM482dg; -C—Negative control 1VM291br; +C—Positive control 1VM118g; +C— (2VM963Lg); 1—2M1090; 2—2VM983br; 3—1VM147g; 4—1VM97yg; 5—3VM566g; 6—2VM902br; 7—1VM83y; 8—3VM566Lg; 9—1VM403g; 10—2VM964yg; 11—1VM132g; 12—1VM408dg; 13—1VM239g; 14—1VM79g; 15—3VM482dg; 16—1VM328g; 17—1VM175g; 18—3VM671g; 19—1VM39wy; 20—2VM966yg; 21—2M1223yg; 22—2VM882g; 23—2VM890g; 24—2VM892g; 25—1VM80sg. (b) *Aspergillus flavus* isolates from Nandi county: M—molecular weight 100 bp ladder; 1—BM69YG; 2-BM38YG; 3—BM73YG; 4—BMG; 5—BM1YG; 6—BM10YG; 7—BMYG

### 3.3. Molecular Analysis of *Aspergillus* Strains and Aflatoxin Production on CAM

**Figure 3:**
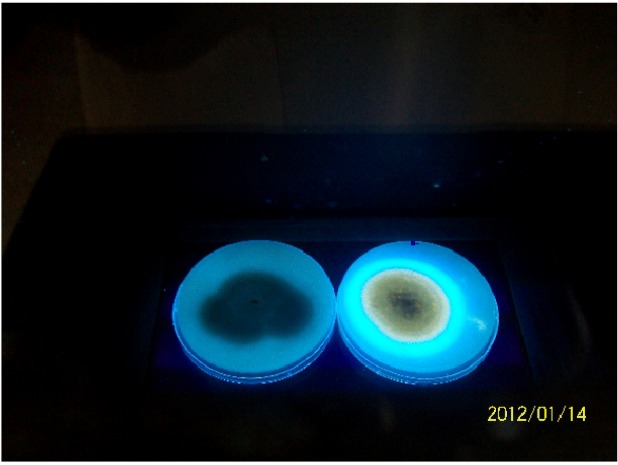
Colonies of non-aflatoxigenic and aflatoxigenic strains of *Aspergillus flavus* observed under ultra violet light. Strains were cultivated in Yeast Extract Sucrose (YES) medium and photographed on the third day of incubation at 28 °C.

Figure 2 is a representative of the electrophoretic band patterns obtained for both *afl*D (400 bps) and *afl*Q (719 bps). All isolates that were positive for any one or both gene amplicons fluoresced on CAM after 3 days of incubation at 28 °C, whereas those that were negative for both *afl*D and *afl* Qamplicons did not fluoresce. Figure 3 shows colonies of non-aflatoxigenic and aflatoxigenic isolates of *A. flavus* observed under UV light. 

**Figure 4:**
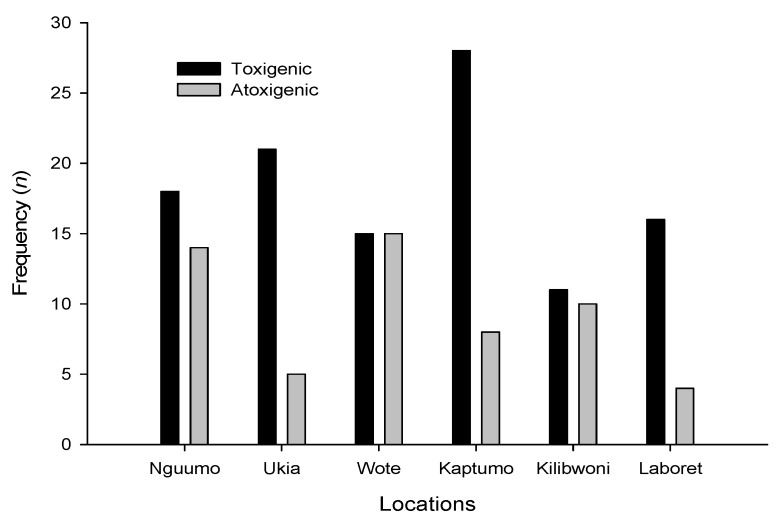
Frequency of toxigenic strains of *Aspergillus flavus* in two zones in Kenya.

Of the 78 *A. flavus* isolates tested in Nandi 71% (*n* = 55) were positive for the aflatoxigenic genes while in Makueni 87 isolates were tested and 62% (*n* = 54) positive. This difference was not significant (χ^2^_0.05(1)_ = 1.2422; *p* = 0.265). Positive (toxigenic) strains were significantly predominant over negative (non-toxigenic) strains in all the locations (Table C; χ^2^_0.05(5)_ = 54.9; *p* < 0.0001). Figure 4 shows the distribution of toxigenic and non-toxigenic isolates within the locations. Ukia, Laboret and Kaptumo had over 70% occurrence of toxigenic isolates compared with non-toxigenic isolates.

Of the seven *A. parasiticus* isolates from Makueni, four expressed the toxigenic genes while in Nandi out of the 22 isolates, four were positive.

### 3.4. Production of Aflatoxins by the Isolates in Vitro

All the 78 isolates with toxigenic genes produced AFB1. Only six did not produce AFB2. AFB1 was produced in the largest quantities (highest of 83491 ppb from Makueni and 64030 ppb from Nandi) followed by AFB2 (highest 52600 ppb from Makueni and 59231 ppb from Nandi), AFG1 (highest 28728 from Makueni and 48914 ppb from Nandi) and lastly AFG2 (highest 8411 ppb from Makueni and 9629 ppb from Nandi). Makueni isolates produced more AFB1 and AFB2 than Nandi isolates while the reverse was true for the G aflatoxins. AFB2 was detected in all isolates except four from Nandi and two from Makueni. Out of the 83 isolates, AFG1 was not detected in 14 samples (11 from Nandi and three from Makueni). AFG2 was detected in only eight isolates (four from Nandi and another four from Makueni). Ten isolates from Nandi and five from Makueni did not produce G aflatoxins. The amount of toxins produced varied with isolates (Table 3).

**Table 3:** Production of aflatoxins *in vitro* by *Aspergillus flavus* and *A. parasiticus *isolates from Makueni and Nandi. Values reading nil (not detected) equal <20 ng

(a) Makueni isolates						
**Code**	**Isolate**	AFB1** (µg/kg)**	AFB2** (µg/kg)**	AFG1** (µg/kg)**	AFG2** (µg/kg)**	**Total Aflatoxin (µg/kg)**
1VM326g	*A. flavus*	8159	2037	2	0	10199
2VM890g	*A.flavus*	19916	9564	1	2	29484
Kp-B	*A. flavus*	1502	343	6	0	1850
2VM983g	*A.flavus*	13143	2265	20	0	15427
2M1204g	*A. flavus*	127	13	0	0	140
Km-A	A. flavus	57	0	5	0	62.133
1VM79g	*A. flavus*	257	75	0	0	332.35
2M1002L	*A.flavus*	63390	33777	17	0	97184
1VM403g	*A.flavus*	10113	3438	22	0	13574
1VM147g	*A. flavus*	898	267	0	0	1165
2M1014g	*A. flavus*	702	82	1	0	785
1VM402g	*A.flavus*	12032	6989	17	0	19038
2VM892g	*A.flavus*	33173	14458	22	0	47653
1VM83y	*A. flavus*	1988	721	203	0	2912
3VM482g	*A.flavus*	14092	8947	30	0	23069
1VM235g	*A. flavus*	459	135	7	0	602
1VM130d	*A.flavus*	61878	30349	22	0	92249
1VM175g	*A.flavus*	52229	15100	20	0	67350
3VM559g	*A. flavus*	1686	200	7	0	1893.2
2M1090g	*A. flavus*	516	50	568	64	11971
1VM223g	*A.flavus*	32145	15245	365	65	47820
2M1016g	*A. flavus*	798	229	12	0	1039
1VM328g	*A.flavus*	31797	6743	54	0	38594
Km-B	*A. flavus*	673	81.356	12	0	766
2VM882g	*A.flavus*	30939	11627	10	0	42576
2M1365g	*A.flavus*	83491	52600	16875	0	152966
3VM551g	*A.flavus*	30637	13620	21	0	44278
Um	*A. flavus*	1692	420	8	0	2120
2M1383g	*A. flavus*	270	47	7	0	324
2M1122g	*A. flavus*	5028	1008	42	0	6078
1VM250	*A. flavus*	2719	969	8	0	3695
1VM95yg	*A.flavus*	59374	29456	25	0	88878
1VM79Lg	*A. flavus*	1073	382	3	0	1458
3VM643g	*A.flavus*	339	47	0	0	387
2VM983g	*A. parasiticus*	22	0	0	0	22
1VM149g	*A.parasiticus*	15139	3141	28728	8411	55419
1VM118g	*A. parasiticus*	97	6	9	0	112
2VM983	*A. parasiticus*	45	8	8	0	61
(b) **Nandi isolates**						
**Code**	**ISOLATE**	AFB1** (µg/kg)**	AFB2** (µg/kg)**	AFG1** (µg/kg)**	AFG2** (µg/kg)**	**Total Aflatoxin ** **(µg/kg)**
BM I YG	*A.flavus*	42	4	0	0	46
BM130YG	*A.flavus*	64030	16583	51	0	80664
BM112YG	*A.flavus*	13775	2054	48914	9624	74367
BM 43 G	*A.flavus*	898	56	7	0	962
BM 12 G	*A.flavus*	35	0	22	0	57
BM 1 G	*A.flavus*	110	38	3	0	151
BM 59 G	*A.flavus*	21	1	0	0	22
BM 1G	*A.flavus*	10164	2622	17	0	12803
BM 3 YG	*A.flavus*	39	8	49	0	96
BM 1 G	*A.flavus*	57142	59231	292	0	116666
BM 3 G	*A.flavus*	23	7	3	0	33
BM 9 G	*A.flavus*	22	0	0	0	22
BM 54 YG	*A.flavus*	12888	9272	44	0	22203
BM 80 G	*A.flavus*	6233	2857	0	0	9089
BM 1 YG	*A.flavus*	91	0	0	0	91
BM 78 YG	*A.flavus*	31	7	4	0	42
BM 1 YG	*A.flavus*	119	25	1	0	145
BM 14 G	*A.flavus*	3771	2488	9826	7798	23883
BM 69 G	*A.flavus*	16	6	0	0	22
BM 1 G	*A.flavus*	40	6	0	0	46
BM 1 YG	*A.flavus*	200	43	4	0	247
BM 1 G	*A.flavus*	10058	1217	17	0	11292
BM 49 G	*A.flavus*	64	10	2	0	77
BM 1 Y	*A.flavus*	108	9	207	0	325
BM 1 G	*A.flavus*	12960	4661	7	0	17628
BM 55 YG	*A.flavus*	13288	8072	54	0	21413
BM 1 YG	*A.flavus*	77	18487	68	0	18632
BM 1 YG	*A.flavus*	2923	25	514	0	3463
BM 2 YG	*A.flavus*	806	488	9	0	1304
BM 3 YG	*A.flavus*	42	13	7	0	63
BM 4 YG	*A.flavus*	216	53	0	0	269
BM 2 G	*A.flavus*	33	8	3	0	44
BM 1 G	*A.flavus*	18	0	0	0	18
BM 1 YG	*A.flavus*	19	2	2	0	24
BM 1 G	*A.flavus*	41	5	0	0	46
BM 2 G	*A.flavus*	139	18	63	0	221
BM 38 YG	*Aparasiticus*	10763	2876	24	0	13662
BM 39 G	*A.parasiticus*	11077	832	1288	0	13197
BM 1 YG	*A.parasiticus*	603	208	1699	7798	10308
BM 1 G	*A.parasiticus*	991	292	5982	2717	9982

Comparative toxin readings from *A. parasiticus* isolates from both regions were not as high as those from *A. flavus.* Isolates from Nandi produced more toxins compared to those from Makueni. 

## 4. Discussion

The results obtained in this study have provided, for the first time, an important comparison of fungal contamination between maize sampled in Makueni County, the main region that has experienced repeated lethal human aflatoxicosis outbreaks and Nandi County, the main maize growing region in Kenya. The study shows that *Aspergillus*, specifically Section *Flavi*, are the main contaminants of maize in household storage in the two regions and *A. flavus* was the most common species. The incidence of occurrence of *A. flavus* in Nandi and Makueni was the same regardless of the differences in mean temperatures (20 °C and 24 °C, respectively) and rainfall (900–1800 and 950–1500 mm). High temperatures and drier conditions are known to favor infection by *A. flavus* but this was not the case in this study. Similar results were observed in Nigeria [25]. 

Toxigenic strains of *A. flavus *were more prevalent than non-toxigenic strains across five out of the six locations. This indicates the risk of aflatoxin poisoning in the event that favorable conditions occur in both regions. The widespread occurrence of the fungus indicates the extent of pre-harvest infection; thus field management strategies stand out as an indispensible intervention strategy towards the fight against aflatoxin contamination of maize in Kenya. In this regard, Abbas *et al.*, [26] observed a higher incidence and greater numbers of *A. flavus* infection and toxin production when there was no crop rotation. Maize is planted in the same fields every season in the two regions. The importance of field management is further stressed by Zablotowicz *et al.*, [27] who report that the history of maize cultivation in terms of soil fertility factors correlates with the occurrence of *A. flavus* and toxin production. 

All the *A. flavus* toxigenic strains from Makueni maize were of the S-type while those from Nandi belonged to the L type. Quantitative and qualitative differences in aflatoxin production *in vitro* between isolates and between these strains were detected. The S strains were confirmed to produce relative larger amounts of total aflatoxins, AFB1 and AFB2 and lower values for AFG1 and AFG2 *in vitro* compared with the L strains. AFB1 is known to be more toxic than the other aflatoxins, and this explains why the S strain has been associated with acute aflatoxin poisoning in Makueni [28]. This is accentuated by high temperatures in Makueni (range 20–28 °C) compared to Nandi (range 18–24 °C), which promotes toxin production [6,29]. However, some L strain isolates from Nandi produced large amounts of AFB1 and AFB2 contrary to the findings of Cotty [13], and Egel [30]. It is important that *in vivo* tests for toxin production with the L strains from Nandi are done to confirm the capability of these isolates to produce large amounts of toxin. These L strains pose a threat of endemic chronic exposure to humans if the maize is exposed to conditions suitable for toxin production given that they are distributed widely in the region. Further, Nandi is the major maize production zone in the country implying that the maize is distributed to most parts of the country. Control of moisture and temperature during transportation and storage is important since the maize is already contaminated with the toxigenic *A. flavus. *The most toxic AFB1 were produced in larger quantities compared to the AFB2. AFG1 were produced in low quantities and only eight out of 78 samples tested produced AFG2. 

This is the first report of *A. flavus* S and L strains isolated from Kenya producing both B and G aflatoxins on YES agar. Probst *et al.*, [3,28] isolated in Kenya both S and L strains, which produced only B aflatoxins on maize. Ehrlich *et al.*, [31] reported that all members of *A. flavus* lack the ability to synthesize G aflatoxins due to a 0.8- to 1.5-kb deletion in the 28-gene aflatoxin biosynthesis cluster agreeing with Cotty and Cardwell [32] and Egel [29]. However, S strains from Benin produced both AFB1 and AFG1 aflatoxins [33]. Davis *et al.*, [34] also observed production of aflatoxin AFB1 and AFG1 by *A. flavus* in a synthetic medium. Cotty and Cardwell [32] reported that SBG strains from the United States and West Africa produced both B and the G aflatoxins. It is not clear if the difference in media is the reason for the variation in the production of B and G toxins or if the isolates that produced both toxins in this study are similar to the SBG strains referred to above. However Abbas *et al.* [35] demonstrated that cultural methods are suitable and effective in screening aflatoxin production by *Aspergillus* isolates. *Aspergillus parasiticus* was rare in both regions. The number of isolates was too small to allow a conclusion on their toxin production ability. The main source of aflatoxin contamination in maize in eastern and western Kenya is *A. flavus*. 

Incidences of sclerotia formation were greater among the S-type than the L-type on V8-juice agar. Sclerotia are important survival structures in the life cycle of many fungi. When conditions are favorable, they germinate into hyphae, which then form conidia. The conidia are blown away by the wind and reinfect maize kernels through the silk. Studies on the conditions responsible for sclerotium initiation might be important to develop methods for suppressing the formation of sclerotia, resulting in reduced survival of the fungus and better disease management [36]. Sclerotia have been associated with aflatoxin production [37]. However Cotty [14] explains that failure of some isolates of *A. flavus* to produce sclerotia on culture media can be due to one of the following: an attenuation of sclerotial production in culture, an unfavorable medium, unfavorable temperature, the differential sensitivity of isolates to light, or other environmental constraints in culture and that strain L isolates require more precise conditions to produce sclerotia than strain S isolates. However, disagreements between studies correlating the sclerotial production of isolates with aflatoxin production exist [37]. 

Apart from the risk of aflatoxin contamination of maize, bio-deterioration is another problem associated with high fungal contamination of kernels [38]. The internal mycoflora of maize in Nandi and Makueni is similar and is dominated by the species of *Aspergillus*, *Fusarium* and *Penicillium, *which predisposes the kernels to bio-deterioration. The resulting physiological and biochemical changes in the maize kernels eventually render grains unsuitable for human consumption. In addition, species of the genera *Fusarium*, *Penicillium* and *Alternaria* were isolated and some species are capable of producing a wide spectrum of compounds shown to be toxic to man and animals [39] and so increase the risk of multi-mycotoxin contamination and exposure. 

About 40% of maize kernel contamination was caused by *Fusarium* and less frequently *Penicillium* and *Alternaria* were also part of the internal mycoflora of maize. *Fusarium* species are the most important mycotoxin producers in northern temperate regions and their presence as part of the internal mycoflora of maize raises a concern. Occurrence of *Fusarium* spp. in maize in Kenya and fumonisin production has been reported previously [40]. Muthomi, *et al.*, [41] reported *Fusarium* as the most predominant species in maize from eastern Kenya and not *Aspergillus,* but this observation may have been influenced by the fact that a *Fusarium* selective media was used for isolation. The frequency of isolation of *Aspergillus*, *Fusarium*, *Penicillium and Alternaria *was the same in the two regions. However, location variation was significant and this may be attributed to differences in pre- and post-harvest management practices, at location and household level. There was a high incidence of *Aspergillus* contamination in Nguumo and Kaptumo. *Fusarium* and *Penicillium* were dominant in Kilibwoni and Ukia. Laboret, a location constituted mostly of medium scale maize producers, recorded the highest incidence of clean maize. Medium-scale farmers are better placed to manage farming and storage of maize than small-scale farmers resulting in reduced aflatoxin contamination.

## 5. Conclusions

This research has shown that *A. flavus* is the main fungal species infecting maize grains in Kenya and that toxigenic strains are widespread. The situation is accentuated in the Eastern Province by the existence of the highly toxigenic S strain and high prevailing temperatures. However, the presence of the L strains should not be overlooked, as this indicates the possibility of chronic exposure from the lower levels of toxins produced by these strains. The influence of aflatoxins on human populations in Kenya over the past decade demonstrates a clear need for tools to manage contamination of locally produced maize. Given the widespread nature of toxigenic strains, any control strategy will have to include field interventions. Fungal infection of maize varies with households and this reflects different farm management practices, some of which adversely contribute to contamination of the maize value chain.
